# Plain cigarette packs do not exert Pavlovian to instrumental transfer of control over tobacco-seeking

**DOI:** 10.1111/add.12756

**Published:** 2014-11-13

**Authors:** Lee Hogarth, Olivia M Maynard, Marcus R Munafò

**Affiliations:** 1 School of Psychology, University of ExeterExeter, UK; 2 School of Psychology, University of New South WalesSydney, NSW, Australia; 3 MRC Integrative Epidemiology Unit (IEU), University of BristolBristol, UK; 4 UK Centre for Tobacco and Alcohol Studies, School of Experimental Psychology, University of BristolBristol, UK

**Keywords:** Cue–reactivity, Pavlovian to instrumental transfer, plain packaging, public health, smoking cessation, tobacco-seeking.

## Abstract

**Aims:**

To gain insight into the potential impact of plain tobacco packaging policy, two experiments were undertaken to test whether ‘prototype’ plain compared with branded UK cigarette pack stimuli would differentially elicit instrumental tobacco-seeking in a nominal Pavlovian to instrumental transfer (PIT) procedure.

**Design, Setting and Participants:**

Two experiments were undertaken at the University of Bristol UK, with a convenience sample of adult smokers (experiment 1, *n* = 23, experiment 2, *n* = 121).

**Measurement:**

In both experiments, smokers were trained on a concurrent choice procedure in which two responses earned points for cigarettes and chocolate, respectively, before images of branded and plain packs were tested for capacity to elicit the tobacco-seeking response in extinction. The primary outcome was percentage choice of the tobacco- over the chocolate-seeking response in plain pack, branded pack and no-stimulus conditions.

**Findings:**

Both experiments found that branded packs primed a greater percentage of tobacco-seeking (overall mean = 62%) than plain packs (overall mean = 53%) and the no-stimulus condition (overall mean = 52%; *P*s ≤ 0.01, ŋ_p_^2^s ≥ 0.16), and that there was no difference in percentage tobacco-seeking between plain packs and the no-stimulus condition (*P*s ≥ 0.17, ŋ_p_^2^s ≤ 0.04). Plain tobacco packs showed an overall 9% reduction in the priming of a tobacco choice response compared to branded tobacco packs.

**Conclusions:**

Plain packaging may reduce smoking in current smokers by degrading cue-elicited tobacco-seeking.

## INTRODUCTION

Studies have shown that in current smokers, plain cigarette packs are less appealing 1, provoke less craving and motivation to purchase 2, reduce short-term self-reported smoking rates 3,4 and increase attention to health warnings 5,6 compared to branded packs. Although these studies suggest that plain packs may reduce smoking motivation, further direct examination of whether plain packs reduce actual tobacco-seeking behaviour is required to gain insight into the potential effectiveness of this policy. In the natural environment, tobacco products arguably act as discriminative stimuli which set the occasion (signal) when instrumental tobacco-seeking is typically reinforced, and thereby come to elicit tobacco-seeking behaviour. From this viewpoint, switching to plain packs may degrade the discriminative control function of pack stimuli, weakening their capacity to elicit instrumental tobacco-seeking 7. The aim of the current experiments was to test this prediction.

The outcome-specific Pavlovian to instrumental transfer (PIT) procedure provides an important assay of the discriminative control function of a stimulus, i.e. its ability to prime instrumental responding for the reinforcer signalled by the stimulus 8,9. The PIT procedure involves training two responses which earn different reinforcers or outcomes. Then, free choice between the two responses is tested in extinction, while stimuli are presented which have been associated previously, and separately, with the same reinforcers. The key finding is that each stimulus selectively augments performance of the congruent response, i.e. the response that earns the same reinforcer as that signalled by the stimulus. As the PIT test is conducted in extinction, stimuli arguably bias choice by interacting with knowledge of the response–outcome contingencies established in the acquisition stage.

Although developed initially as an animal model with natural rewards 8, the PIT procedure has now been used extensively with humans (e.g. 9) using tobacco 10–15 and alcohol cues as rewards 16–18 to model drug cue–reactivity, i.e. to study how drug cues provoke instrumental drug-seeking. In the tobacco version of the PIT procedure, smokers are first trained on a concurrent choice procedure in which one response earns tobacco points (which participants believe are exchangeable for a pack of cigarettes present on the table), whereas the alternative response earns chocolate points (notionally exchangeable for a chocolate bar, also present). Following this acquisition stage, participants are tested for free choice between the two responses in a nominal extinction stage where earned points are not displayed to participants until later. Various types of smoking cues have been presented in this PIT test to evaluate their capacity to prime selection of the tobacco-seeking response, including smoking-related images 11–13,15, abstract discriminative stimuli that have previously signalled that a different response would be reinforced with tobacco points 14 and abstract Pavlovian conditioned stimuli that have previously signalled the presentation of tobacco points 16, compared to control cues associated with chocolate. It has been found that each stimulus selectively biases choice in favour of the response that earns the same outcome as that associated with the stimulus. This ability of tobacco stimuli to increase free choice of the tobacco-seeking response potentially models the motivating effect of smoking cues in the natural environment.

Insight into the psychological basis of the PIT effect comes from several observations. First, the PIT effect is greater for discriminative stimuli which signal when a response will be reinforced than for Pavlovian conditioned stimuli which signal the occurrence of the reinforcer 7,19,20. Relatedly, the PIT effect can be abolished more readily by discriminative extinction training where the stimulus signals the non-reinforcement of a response than by Pavlovian extinction training where the stimulus signals the non-occurrence of the reinforcer 16,21–24. Moreover, the human PIT effect is correlated with participants’ self-reported expectations that PIT stimuli signal which response (of two) is more likely to be reinforced, and can be abolished by simply telling participants that this is not the case 16. These data are consistent with the claim that PIT cues prime response choice by enhancing the expectation that the response which earns the signalled reinforcer is more likely to be reinforced, i.e. the PIT effect is propositional in nature (25; but see 26).

By contrast, the tobacco PIT effect is not related to individual differences in tobacco dependence level 11–13, consistent with other cue–reactivity paradigms 27. Furthermore, the tobacco PIT effect is not modulated by changing the current value of smoking through deprivation/satiety 11, smoking health warnings 11, nicotine replacement therapy 12 or, using a slightly different cue–reactivity paradigm, varenicline 28. In animals, too, the PIT effect is not modulated by changing the value of the signalled outcome (e.g. 29). The implication is that smoking stimuli enhance smokers’ beliefs that tobacco-seeking will be reinforced, which raises the propensity to select this response by a constant, irrespective of how valuable smoking currently is to the individual. This form of stimulus control over tobacco-seeking is paradoxical, being propositional in nature yet autonomous of the individual's desires, and is precisely the form of stimulus control one would expect to produce drug use and relapse despite intentions to remain abstinent. The purpose of the current experiments was to test whether this form of stimulus control over tobacco-seeking would be weaker in plain versus branded cigarette packs.

## EXPERIMENT 1

**Figure 1:**
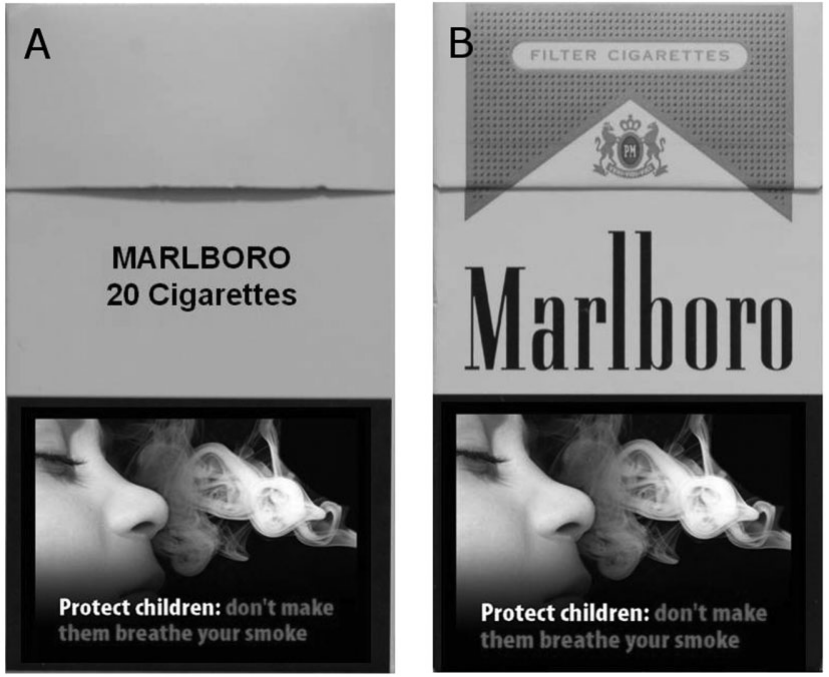
Examples of the cigarette pack stimuli presented on-screen prior to choice between the tobacco- versus chocolate-seeking response in the Pavlovian to instrumental transfer (PIT) test: (a) plain pack; (b) branded pack

Experiment 1 used the previously described PIT procedure to test whether plain cigarette pack stimuli would show reduced control over tobacco-seeking than branded pack stimuli. Smokers were first trained on a concurrent choice task in which one response earned tobacco points for a branded cigarette pack, whereas the alternative response earned chocolate points. In the PIT test that followed, choice between the two responses was tested in extinction during presentation of either an image of a plain pack (Fig. 1a, from 5,6) or a branded UK pack (Fig. 1b). Blank no-stimulus trials were intermixed randomly. It was expected that whereas branded pack stimuli would augment the tobacco-seeking response relative to no-stimulus trials consistent with previous data 11–13,15, plain pack stimuli may show reduced capacity to elicit the tobacco-seeking, demonstrating their degraded discriminative control.

## METHOD

### Participants

Smokers (*n* = 24) were recruited from the staff and students at the University of Bristol. Half were regular daily smokers (50% male), defined as smoking every day of the week at least five cigarettes per day starting within 1 hour of waking, and half were non-daily smokers (50% male), defined as smoking at least one cigarette per week, but not daily. Participants were sampled in this way to ensure a normal distribution of individuals across the continuum of smoking heaviness indexed in these studies by cigarettes smoked on smoking days. All participants were asked to abstain overnight from smoking prior to completing the study, and compliance was checked with breath carbon monoxide (CO) measurement and self-report. Participants were debriefed and reimbursed £5 for their time and expenses, and ethical approval was granted by the University of Bristol, Faculty of Science Research Ethics Committee. We calculated the required sample size for experiment 1 to demonstrate a PIT effect produced by the branded cues, on the assumption that the effect size would be similar to that observed for the cigarette cues used in a previous published study 13. This effect size (*dz* = 0.79) indicated that a sample size of *n* = 24 would provide 95% power at an alpha level of 5%.

### Procedures

Informed consent was obtained immediately prior to the screening procedure comprising breath alcohol concentration (BrAC) and exhaled CO readings, and assessment of eligibility based on exclusion criteria: self-reported alcohol consumption with 24 hours, cigarette smoking within 8 hours, current use of illicit drugs, current or past medical or psychiatric illness or clinically significant abnormality.

#### Concurrent choice acquisition

Participants completed the concurrent choice acquisition phase of the computer task. The aim of this task was to establish two instrumental responses (key presses) which earned tobacco or chocolate points, respectively. Participants were presented with the rewards: one sealed packet of 10 cigarettes of their preferred UK brand and two sealed 49-g Cadbury Dairy Milk chocolate bars, placed on the table above the keyboard in the spatial position concordant with the key that earned that reward. Participants were informed that they could earn these items in the computer task to take away at the end (however, this was a deception and they did not receive these rewards for ethical reasons).

On-screen instructions for concurrent choice acquisition stated: ‘This is a game in which you can win the cigarettes and chocolate in front of you. In each trial, hold down the D or H key to see if you have won a point for these rewards. You will only win on some trials. Press the space bar to begin’. Each trial began with the centrally presented text, ‘Select a key’, which remained until either the ‘D’ or ‘H’ key was pressed. Pressing one key immediately presented the outcome text, ‘You win one tobacco point’, accompanied by a picture of two cigarettes on a white background, whereas pressing the other key produced the outcome text, ‘You win one chocolate point’, accompanied by a picture of a Cadbury Dairy Milk chocolate bar on a white background, to signal the reward earned by the response. The response–outcome assignment was counterbalanced between participants. Each key had only a 50% chance of yielding its respective outcome. On non-rewarded trials, the outcome text, ‘You win nothing’, was presented. These outcomes were presented for the duration for which the key was held down. Following release of the key, a random intertrial interval between 750 and 1000 ms elapsed prior to the next trial. There were 40 trials of concurrent choice acquisition training in total. At the end, participants were tested for knowledge of the response–outcome contingencies via on-screen questions: ‘Which key earned tobacco/chocolate, the D or the H key? Please choose carefully’. The order of the two questions was randomized. Data from the acquisition phase were not analysed because the study was focused on the magnitude of the PIT effect in the test that followed (overall percentage of tobacco choice in acquisition and the PIT test were equivalent, *F* < 1).

#### PIT test

Prior to the PIT test, participants received instructions about the nominal extinction schedule: ‘In this part of the task, you can earn cigarettes and chocolate by pressing the D or H keys in the same way as during the first part of the experiment. However, you will only be told how many points you have earned for each reward at the end of the experiment. Press the space bar to begin’. The purpose of this arrangement was to ensure that responding remained stable while the impact of cues was assessed. Participants then completed the transfer test in which they chose between the tobacco and chocolate keys (in extinction) in the presence of no stimulus, a plain pack (Fig. 1a, from 5,6 or a branded UK pack (Fig. 1b). These PIT stimuli were presented concurrently with the prompt 'Select a key' until the D or H key was selected. There were 60 PIT trials in total, blocked into 10 cycles of six trials, where two each of the three cue conditions (no-stimulus, plain, branded) were presented in random order. In trials where a pack stimulus was presented, the pack was sampled randomly from a set of 100 stimuli (10 brands × 10 health warnings). The outcome examined was whether the plain versus branded packs would differ in their capacity to elicit the tobacco-seeking response.

### Data analysis

Percentage choice of tobacco over chocolate was contrasted between the plain pack, branded pack and no-stimulus condition of the PIT test, in a within-subjects analysis of variance (ANOVA). Correlations were conducted with cigarettes smoked on smoking days against overall percentage of tobacco-seeking in the PIT test, and the enhancement of tobacco-seeking by the branded and plain pack stimuli over the no-stimulus condition. All variables were normally distributed. Exact *P*-values are reported throughout.

### Results and discussion

**Figure 2:**
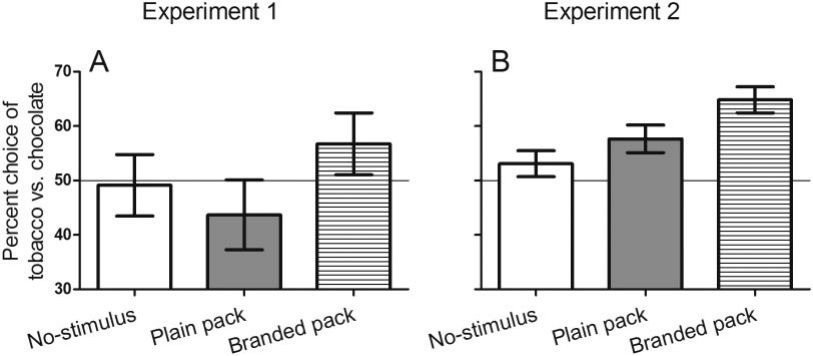
The vertical axis shows the mean percentage choice of the tobacco- versus chocolate-seeking response [± standard error of the mean (SEM)] during the Pavlovian to instrumental transfer (PIT) test (50% = equal choice or indifference). The horizontal axis shows the stimulus that was presented on the screen before a response choice was made: either no-stimulus, an image of a plain pack (Fig. 1a) or a branded pack (Fig. 1b). In both experiments, the branded pack stimulus primed tobacco-seeking more than the no-stimulus and plain pack conditions, and the latter two conditions did not differ

One participant was excluded for reporting inaccurate knowledge of the response–outcome contingencies following concurrent choice acquisition, leaving a final sample of *n* = 23 for analysis. These participants had a mean age of 20.8 years [standard deviation (SD) = 2.3, range = 18–27] and smoked an average of 9.3 cigarettes on smoking days (SD = 5.7, range = 1–25). Table 1 and Fig. 2a show the percentage choice of the tobacco- versus the chocolate-seeking response in the three stimulus conditions of the PIT test. ANOVA with these data (see Table 2) produced a main effect of stimulus (*F*_(2,44)_ = 3.44, *P* = 0.04, ŋ_p_^2^ = 0.14). Crucially, that there was no evidence of a difference in tobacco-seeking between the plain pack and the no-stimulus condition (*F*_(1,22)_ = 0.97, *P* = 0.33, ŋ_p_^2^ = 0.04). This critical null result was confirmed by a Bayes factor of 0.38, indicating low confidence in this difference. Accordingly, the plain and no-stimulus conditions were averaged, and the branded pack enhanced tobacco-seeking above this average (*F*_(1,22)_ = 7.23, *P* = 0.01, ŋ_p_^2^ = 0.25). The failure of the plain packs to elicit tobacco-seeking in the PIT test, in contrast to branded packs, demonstrates that the discriminative control function of plain packs is degraded.

**Table 1:** Mean percentage choice of the tobacco- versus chocolate-seeking response (standard error of the mean) during the Pavlovian to instrumental transfer (PIT) test of experiment 1, in the presence of no-stimulus, an image of a plain pack (Fig. 1a) or an image of a branded pack (Fig. 1b)

Experiment 1		
No-stimulus	Plain pack	Branded pack
49.1 (5.6)	43.7 (6.4)	56.7 (5.6)

**Table 2:** Analysis of variance (ANOVA) on the Pavlovian to instrumental transfer (PIT) data of experiment 1 in Table 1

	dƒ	F	P	ŋ_p_^2^
Stimulus	2,44	3.44	0.04*	0.14

* Significant.

The number of cigarettes smoked on smoking days correlated with the overall preference for choosing the tobacco over the chocolate response in the PIT phase (*r* = 0.54, *P* = 0.01), but not with the magnitude of the PIT effect above the no-stimulus condition produced by the plain packs (*r* = −0.29, *P* = 0.17) or branded packs (*r* = −0.09, *P* = 0.66), consistent with previous findings 11–13.

## EXPERIMENT 2

The purpose of experiment 2 was to test whether the failure of plain pack stimuli to elicit tobacco-seeking in the PIT test, found in experiment 1, could be replicated in a larger sample. To this end, the procedure of experiment 1 was included in the test phase of a larger randomized controlled trial (RCT) 30. One group in this RCT had smoked *ad libitum* for 24 hours from their usual branded UK cigarette pack, and this group received an identical PIT procedure to experiment 1. By contrast, the other group in the RCT had smoked *ad libitum* for 24 hours from a plain Australian cigarette pack, and for this group, the Australian plain cigarette pack served as the reinforcer for the tobacco-seeking response in the PIT procedure, but otherwise the PIT procedure was identical. The prediction was that plain compared to branded pack stimuli would fail to elicit tobacco-seeking in both groups, replicating experiment 1 and demonstrating the reliability of plain packs’ degraded discriminative control.

### Method

Experiment 2 reports part of the test phase of a randomized controlled trial, the full protocol for which has been registered (ISRCTN 52982308) 30. Briefly, 128 cigarette smokers (50% males per group) who smoked between five and 20 cigarettes a day every day of the week, and smoked within 1 hour of waking, were screened and assigned to smoke either a plain Australian pack or their usual branded UK pack of 20 cigarettes *ad libitum* for a full day. Participants were regular smokers of one of the following brands, which are available in both countries: Marlboro Gold, Marlboro Red, Dunhill Red, Benson and Hedges Gold and Benson and Hedges Silver. Self-reported smoking and inhaled volume determined by smoking topography during the 24-hour period were the primary outcome measures of the RCT. Upon returning, participants completed a battery of questionnaires to determine smoking rates and quit intentions, etc. and, finally, completed a task identical to experiment 1. The only difference was that the cigarette pack placed on the table, which participants earned points towards, was the same as the one that participants had smoked during the preceding 24 hours; that is, either a plain Australian pack or branded UK pack of 20 cigarettes of their preferred brand (recall that in experiment 1, participants preferred branded the UK pack of 10 cigarettes served as the tobacco reinforcer). The alternative reward was two 49-g bars of Cadbury Dairy Milk chocolate identical to experiment 1. The tobacco reinforcer was the pack smoked during the *ad-libitum* period to ensure that subsequent tests conducted on the two groups, which were relevant to the RCT, were not affected by the reinforcer used in the PIT test. We were not interested in differences in the PIT effect between the two groups, given the complexity of interpreting any such differences. At the end of the experiment, participants were debriefed and reimbursed £30 for their time and expenses. Ethical approval was granted by the University of Bristol, Faculty of Science Research Ethics Committee.

### Results and discussion

Data analysis followed the same model as experiment 1, except that the between-subjects factor, group (branded pack, plain Australian pack), was added. Seven participants were excluded due to computer failure or inaccurate knowledge of the response–outcome contingencies, leaving a final sample of *n* = 121 for analysis. These participants had a mean age of 21.3 years (SD = 3.32, range = 18–36) and smoked an average of 10.1 cigarettes per day (SD = 3.0, range = 5–17), which did not differ between groups (*P*s > 0.19). Table 3 shows the percentage choice of the tobacco- versus the chocolate-seeking response in the three stimulus conditions of the PIT test for the two groups (Fig. 2b shows the stimulus effect collapsed across group). ANOVA of these data (Table 4) produced a significant main effect of stimulus (*F*_(2,238)_ = 8.60, *P* < 0.001, ŋ_p_^2^ = 0.07, see Fig. 2b). There was no evidence of a main effect of group (*F*_(1,119)_ = 0.001, *P* = 0.98, ŋ_p_^2^ = 0.00) or of a stimulus × group interaction (*F*_(2,238)_ = 0.19, *P* = 0.83, ŋ_p_^2^ = 0.002). Further examination of the main effect of stimulus indicated that the plain pack stimulus did not elicit greater tobacco-seeking than the no-stimulus condition, (*F*_(1,120)_ = 1.92, *P* = 0.17, ŋ_p_^2^ = 0.02). This critical null result was confirmed by a Bayes factor of 0.04, indicating low confidence in this difference. Accordingly, the plain and no-stimulus conditions were averaged, and the branded pack enhanced tobacco-seeking above this average (*F*_(1,120)_ = 22.22, *P* < 0.001, ŋ_p_^2^ = 0.16). The failure of the plain pack stimulus to elicit tobacco-seeking, in contrast to the branded pack stimulus, replicates experiment 1 in a larger sample.

**Table 3:** Mean percentage choice of the tobacco- versus chocolate-seeking response (SEM) during the Pavlovian to instrumental transfer (PIT) test of experiment 2, in the presence of no-stimulus, an image of a plain pack (Fig. 1a) or an image of a branded pack (Fig. 1b). For the two groups, either a branded UK cigarette pack or plain Australian cigarette pack had been smoked *ad libitum* for the previous 24 hours and served as the reinforcer for the tobacco-seeking response. Figure 1b shows the significant main effect of stimulus collapsed across group

Group	No-stimulus	Plain pack	Branded pack
Branded	53.8 (3.4)	56.6 (3.5)	65.0 (3.6)
Plain Australian	52.4 (3.4)	58.6 (3.7)	64.7 (3.3)

**Table 4:** Analysis of variance (ANOVA) on the Pavlovian to instrumental transfer (PIT) data of experiment 2 in Table 3

	dƒ	F	P	ŋ_p_^2^
Stimulus	2,238	8.60	0.001*	0.07
Group	1,119	0.001	0.98	0.00
Stimulus by group	2,238	0.19	0.83	0.002

* Significant.

The number of cigarettes smoked on smoking day correlated with overall tobacco-seeking in the PIT test (*r* = 0.26, *P* = 0.004), but not with the magnitude of the PIT effect above the no-stimulus condition produced by the plain pack stimulus (*r* = 0.13, *P* = 0.15) or the branded pack stimulus (*r* = 0.01, *P* = 0.89), consistent with experiment 1.

## GENERAL DISCUSSION

Both experiments found that whereas the branded pack stimuli enhanced tobacco choice, consistent with previous findings 11–13,15, the plain pack stimuli did not. Averaging across the two experiments, branded packs primed greater tobacco choice (62%) than plain packs (53%) and the no-stimulus condition (52%), which were in turn not different. Thus, plain tobacco packs produced an overall 9% reduction in the priming of tobacco choice compared to branded tobacco packs. These findings indicate that plain packs have degraded discriminative control over instrumental tobacco-seeking. The key questions are: why did branded packs prime tobacco-seeking, and why did the plain packs fail to do so? Regarding the first question, one cannot argue that the branded packs elicited tobacco-seeking by evoking a general appetitive motivational state, because such a state would have enhanced both the tobacco and the chocolate response equally 31. Moreover, it is difficult to argue that branded packs controlled tobacco-seeking by evoking a motivational state or memory of the current reward value of smoking, because the PIT effect is autonomous of smoking satiety 11, health warnings 11 and nicotine replacement therapy 12. Finally, it is unlikely that branded pack stimuli formed a direct stimulus–response or habitual association with the tobacco-seeking response, because the PIT test was conducted in extinction precisely to avoid contingent reinforcement of that response in the presence of pack stimuli (but see 26). We favour the view that branded packs controlled tobacco-seeking by evoking a specific expectation that the tobacco-seeking response had a greater probability of producing the tobacco outcome 16,25. The basis for this claim is the recent finding that the drug PIT effect is correlated positively with participants’ self-reported expectations that PIT stimuli signal which response will be reinforced, and can be abolished by telling participants that this is not the case 16. The implication is that plain pack stimuli failed to elicit tobacco-seeking because they failed to generate a belief that the tobacco-seeking response was more likely to be rewarded (this remains to be formally tested).

Generalization decrement is the most obvious explanation for why plain pack stimuli failed to elicit tobacco-seeking 32. From this viewpoint, plain packs failed to elicit tobacco-seeking because they are different from the branded packs that participants have learned as signals for tobacco-seeking in the past. One issue for this position, however, is that plain and branded packs (Fig. 1a,b) have many elements in common (size, shape, name, contents, health warnings), and this similarity should have allowed plain packs to exert at least some degree of control over tobacco-seeking (rather than none). In support of this, previous studies have shown that brand cues are not essential to produce a PIT effect 11–13,15; rather, an image showing two non-branded cigarettes alone on a white background produced the same magnitude of PIT effect as the branded pack stimuli used in the current experiments. Why can branded packs and isolated cigarettes produce a PIT effect whereas the plain packs cannot? One possibility is that in the natural environment, although plain pack elements are as associated with tobacco-seeking as brand cues, they are suppressed in their discriminative control over tobacco-seeking by a processes of cue-competition; namely, overshadowing 33,34. From this viewpoint, more salient brand cues ‘out-compete’ plain pack elements for control over tobacco-seeking. Support for this claim comes from studies showing that attention to background elements of pack stimuli is reduced when brand cues are present, and increased when brand cues are absent 5,6 (see also 35). Furthermore, smoking cues can overshadow learning about neutral stimuli which are equally reliable as signals for when a response will be reinforced 36, due presumably to their attentional salience 37. This analysis proposes that by virtue of being the more attentionally salient, brand stimuli overshadow (suppress) learning about plain pack elements as signals for tobacco-seeking, thus abolishing the discriminative control over tobacco-seeking by plain pack elements, which we observed in two studies. The therapeutic question is how long such suppression of discriminative control by plain elements would last following smoking from plain packs in the absence of brand cues when cue-competition is no longer in force.

Another complementary explanation for why plain packs failed to elicit tobacco-seeking appeals to the concept of stimulus–outcome congruity 38. From this viewpoint, pack stimuli presented in the PIT test elicit tobacco-seeking to the extent that they retrieve a representation of the reinforcer earned by that response. As the reinforcer for tobacco-seeking was points for branded UK pack cigarettes (in experiment 1, and the branded group of experiment 2), one would expect branded UK pack stimuli to more readily retrieve a representation of that reinforcer than plain pack stimuli, and thus elicit tobacco-seeking to a greater extent. One slight contradiction to this claim, however, is that the branded pack stimuli produced the same magnitude of PIT effect when the reinforcer for tobacco-seeking was points for the perceptually incongruous plain Australian pack (in the plain Australian group of experiment 2). Here, the presented stimulus and the reinforcer were perceptually different, so one might anticipate a weaker PIT effect, which was not found. Nevertheless, it remains plausible that branded packs exert particular control over tobacco-seeking compared to plain packs, not only because they overshadow other discriminative stimuli 36, not only because they are perceptually congruous with the outcome earned by tobacco-seeking 39, but also because they are emotionally congruous (in being positively valenced) with the rewarding state expected from smoking 40. However, all these potential components of the tobacco PIT effect remain to be fully explored.

It is worth noting that 24 hours of *ad-libitum* smoking from Australia plain packs by half the participants in experiment 2 did not enhance the capacity of plain pack stimuli to elicit tobacco-seeking. This observation might suggest that impaired discriminative control by plain pack stimuli may be somewhat durable following a switch by current smokers to smoking from plain packs. However, this claim is weakened because the Australian plain packs that were smoked from were dark olive green, whereas the ‘prototype’ plain pack stimuli presented in the PIT test were beige (Fig. 1a). Thus, any learning about plain Australian packs during *ad-libitum* smoking may not have generalized to the plain pack stimuli used in the PIT test. Future studies should overcome this limitation by evaluating changes in the PIT effect produced by plain packs following uptake of *ad-libitum* smoking from identical plain packs. Such a study would provide insight into the potential long-term efficacy of the plain packaging policy for current smokers.

To conclude, two experiments found that whereas branded pack stimuli elicited tobacco-seeking in a Pavlovian to instrumental transfer protocol, plain pack stimuli did not, indicating that plain packs have degraded discriminative control over tobacco-seeking. The implication is that plain packaging policy may reduce smoking motivation in current smokers through this mechanism. One bold extrapolation is that because plain packs elicited the tobacco-seeking response on average 9% less than branded packs, one might expect plain packaging policy to reduce current smokers’ consumption of cigarettes by 9%. However, many unknowns complicate this direct extrapolation; for example, what is the relationship between tobacco-seeking and actual consumption, how will the discriminative control of plain packs change with experience, will broader contextual smoking cues moderate the effect of packaging cues, etc.? Thus, although our studies support the view that current smokers’ motivation to smoke will be primed less by plain packaging than branded packaging, the ultimate magnitude of this effect remains to be seen. A secondary finding in experiment 2 was that plain packs’ degraded discriminative control over tobacco-seeking survived 24 hours of *ad-libitum* smoking from somewhat similar Australian plain packs. A key question for future research is how long plain packs’ degraded discriminative control would last following experience of smoking from identical plain packs.

### Declaration of interests

None.
